# Expression of the TGF-beta1 system in human testicular pathologies

**DOI:** 10.1186/1477-7827-8-148

**Published:** 2010-12-02

**Authors:** Candela R Gonzalez, María E Matzkin, Mónica B Frungieri, Claudio Terradas, Roberto Ponzio, Elisa Puigdomenech, Oscar Levalle, Ricardo S Calandra, Silvia I Gonzalez-Calvar

**Affiliations:** 1 Instituto de Biología y Medicina Experimental (CONICET), Vuelta de Obligado 2490 (1428) Buenos Aires, Argentina; 2 División Endocrinología, Hospital Durand, Av. Díaz Velez 5044 (1405) Buenos Aires, Argentina; 3 Instituto Medico PREFER, Calle 995 N° 2348, San Martín (6000) Argentina; 4 Facultad de Medicina, Universidad de Buenos Aires, Paraguay 2155 (1121) Buenos Aires, Argentina

## Abstract

**Background:**

In non-obstructive azoospermia, histological patterns of Sertoli cell-only Syndrome (SCO) and hypospermatogenesis (H) are commonly found. In these pathologies, Leydig cell hyperplasia (LCH) is detected in some patients. Since TGF-β1 is involved in cellular proliferation/development, the aim of this work was to analyze the expression of TGF-β1, its receptors TGFBRII, TGFBRI (ALK-1 and ALK-5), and the co-receptor endoglin in human biopsies from patients with idiopathic infertility.

**Methods:**

Specific immunostaining of TGF-β1, its receptors TGFBRII, TGFBRI (ALK-1 and ALK-5), co-receptor endoglin and Smads proteins, were carried out in testicular biopsies from normal and infertile men with SCO or H. Gene expression of TGF-β1 system were made in biopsies from infertile patients with semi-quantitative and quantitative PCR.

**Results:**

Immunohistochemical studies revealed that TGF-β1 and its specific receptors are present in Leydig cells in biopsies from normal tissue or patients with SCO or H with or without LCH. Smad proteins, which are involved in TGF-β1 signaling, are also detected in both their phosphorylated (activated) and dephosphorylated form in all samples *TGF-β1, ALK-1 *and *endoglin *gene expression are stronger in human biopsies with LCH than in those with SCO or H. Neither *TGFBRII *nor *ALK-5 *gene expression showed significant differences between pathologies. A significant correlation between *ALK-1 *and *endoglin *expression was observed.

**Conclusions:**

In conclusion, the high levels of mRNA and protein expression of the TGF-β1 system in patients with LCH, particularly *ALK1 *and its correlation with *endoglin*, suggest that these proteins acting in concert might be, at least in part, committed actors in the Leydig cell hyperplasia.

## Background

Primary testicular failure affects approximately 1% of all men and 10% of those who seek fertility evaluation. The most common histological patterns found in testicular biopsies of azoospermic men (non-obstructive azoospermia) are either Sertoli cell-only Syndrome (SCO) or hypospermatogenesis (H) [1]. It has been reported that in some of these patients, Leydig cells show hyperplasia (LCH). Two different types of LCH have been described: nodular and diffuse LCH [2]. Identification and characterization of the factors involved in this alteration might be a clue on the understanding of the source of this pathology and could bring new insights into the therapeutic approach.

In experimental animals, LCH may be induced by a number of hormones, enzymes and chemicals [3]. In this context, some reports have indicated that LH, androgens and/or even estrogens might stimulate the division of Leydig cells [4,5]. Leydig cell function is predominantly controlled by gonadotropins. However, a number of intratesticular factors are known to influence Leydig cell steroidogenic function [6]. Among these factors, TGF-β1 modulates Leydig cell steroidogenesis [7] and proliferation [8,9]. In this context, Sanderson et al. [10] have demonstrated that, in transgenic mice overexpressing *TGF-β1*, Leydig cells are prominent.

In several tissues, the TGF-β family has a critical role in the regulation of cell cycle progression and differentiation, in both normal and pathological conditions [11-13].

In the testis, TGF-β regulates a variety of cellular processes, including the secretory function of Leydig and Sertoli cells, as well as the organization of peritubular myoid cells, testis development and spermatogenesis [13-15]. In addition, the absence or overexpression of this factor affects male reproductive function, supporting the hypothesis that TGF-β signaling pathways are carefully coordinated during testicular development and spermatogenesis [13,16,17].

TGF-β1 exerts its effects via specific type I and II serine/threonine kinase receptors. Type II receptor (TGFBRII) transphosphorylates and activates type I receptor (TGFBRI), which then phosphorylates and activates its downstream nuclear effectors, named Smads [12]. It has been described that TGF-β1 activates two distinct type I receptors: activin receptor-like kinase-1 (ALK-1), which promotes Smad 1/5 phosphorylation, and ALK-5, which induces Smad 2/Smad 3 phosphorylation [18]. Activation of ALK-1 stimulates cell proliferation and migration, whereas activation of ALK-5 inhibits these responses. Endoglin, a TGF-β1 co-receptor, is thought to modulate TGF-β1 responses leading to cell proliferation [19]. In endothelial cells, endoglin blocks TGF-β1 induced growth arrest by promoting ALK-1 signaling, and indirectly reduces ALK-5 signaling [20]. Although the presence of TGF-β1, TGFBRII and TGFBRI has been described in human testis [21,22], to our knowledge, there are no reports on the differential expression of the TGFBRI subtype or its co-receptor.

Thus, the aim of the present study was to analyze mRNA and protein expression of TGF-β1, its specific receptors TGFBRII, ALK1 and ALK5, and co-receptor endoglin, in patients with SCO and H and LCH, and to study the presence of Smads 2/3 and 1/5 in these pathologies.

## Methods

### Patients and testicular samples

The study was designed in accordance with the Helsinki Declaration and its last modification in Tokyo 2004 on human experimentation and was approved by The Ethical Committee of the Durand Hospital (Buenos Aires, Argentina) and Ethical Committee of the Instituto de Biología y Medicina Experimental (Buenos Aires, Argentina). Informed Consent was obtained from all the patients enrolled in the study.

Nineteen men (27-42 years-old) presenting idiopathic infertility and non-obstructive azoospermia without infection process were enrolled in this study and were assessed and diagnosed by open testicular biopsy. Patients with known etiology of infertility, such as genitourinary infections, mumps orchitis, varicocele, hypogonadotropic hypogonadism, chromosome anomalies, obstruction or agenesia of seminal ducts were excluded.

The diagnosis of azoospermia was based on the analysis of at least two semen samples collected at different times and two replicates of each sample were centrifuged at 3000 × *g *for 15 min. All the patients underwent a detailed physical examination, endocrinology profile testing and a testicular biopsy. All of them presented normal cariotype. Non-obstructive azoospermia was defined by the presence of spermatogenic defects in the testicular biopsy without evidence of obstruction of the seminal ducts.

All the patients underwent diagnostic testicular biopsy or sperm retrieval and agreed to provide a small piece of testicular tissue (5 mm in diameter) for further studies. One-third of the tissue volume was immersed in Bouin's solution and subjected to histopathological diagnosis. The testicular histopathology was categorized according to the most advanced presence of spermatogenesis. Two pathologists evaluated all testicular slices. Biopsies were classified either as H or SCO appearance, according to McLachlan et al.[23]. The H group included men in which the biopsy specimens contained less than 17 mature spermatids per tubule [24,25]. In addition the presence or absence of LCH in H and SCO sections was recorded. In the LCH group, there were more than 7 Leydig clusters of more than 15 cells per 3.14 mm^2^, in four fields of vision with a 20 × objective [26].

As a consequence, biopsies were classified as SCO (n = 6), SCO + LCH (n = 2), H (n = 8) and H+LCH (n = 3) according to testicular morphology.

Two-thirds of the volume of pathological samples was cryopreserved in liquid nitrogen for molecular biology studies.

Since ethical and legal considerations hampered the possibility to obtain testicular samples from healthy men, histochemistry and studies of molecular biology in normal tissue were carried out from retrospective samples kept in paraffin blocks from patients undergoing bilateral orchiectomy for the treatment of prostate carcinoma (Normal Group (N), n = 4; 50-60 years old). Biopsy specimens from N presented a normal number of mature spermatids per tubule, i.e. more than 17 cells per tubular cross sections [24,25].

### Hormone levels

Before biopsies, a blood sample was taken from patients. Serum concentrations of FSH (normal range 1.5-7 mIU/ml), LH (normal range: 1.1-9 mIU/ml) and testosterone (normal range: 10-30 pmol/ml) were measured by Chemiluminescence's assays (Elecsys 1010, Roche**)**. The intra-and interassay coefficient of variation were 2.1% and 7.5% for FSH, 2.5% and 9.5% for LH, and 5.1% and 8.2% for testosterone respectively. The detection limits were 0.06 and 0.05 mIU/ml for FSH and LH, and 0.35 pmol/ml for testosterone.

### Immunohistochemical staining of testicular tissue

Bouin's fixed human testicular specimens were dehydrated, embedded in paraffin and sectioned at 5 μm. Hematoxylin/eosin stain was used to analyze testicular morphology.

The sections were deparaffinized with 100% xylene and sequentially rehydrated with 96, 90, 80 and 70% ethanol. The slides were then blocked with 3% hydrogen peroxide in absolute methanol for 5 min, washed twice with phosphate-buffered saline (PBS, pH 7.4) for 5 min each time. The sections were subjected to saponine (0.5%) and proteinase K (20 μg/ml) treatments. Nonspecific binding was blocked with normal serum blocking buffer for 20 min at room temperature. The slides were incubated over night with the primary antibody (from Santa Cruz: anti-TGF-β1, sc-146 (1:100); anti TGFβ RI (V-22), sc-402, (1:100); anti TGFβ RI (T-19), sc-398 (1:100); p-Smad 2/3, sc 11769 (1:50); Smad 1/5 sc-6031-R (1:100); endoglin (MJ7/18) sc-18893 (1:50); from Cell Signaling: Smads 2/3 # 3102 (1:100); p-Smad 1/5 #9511 (1:100). 3β-Hydroxy-steroid dehydrogenase (3β-HSD) antibody (1/2000) was kindly provided by Dr. J. Ian Mason (Edinburgh University, United Kingdom).

After five rinses in PBS, sections were incubated for 1 h at room temperature with a biotinylated secondary antibody (Vector Labs, 1:1000). After further washing in PBS, sections were incubated for 1 h with the streptavidin-peroxidase complex diluted 1:100 (ABC kit, Vector Labs). The sections were washed twice with PBS and development of peroxidase activity was performed with 0.055% w/v 3,3'-diaminobenzidine and 0.1% v/v H_2_O_2 _in Tris-HCl. Sections were then washed with distilled water. Negative controls were processed simultaneously by omitting the primary antibodies and were treated with preimmune serum.

The intensity of TGF-β1 and ALK-1 immunostaining was evaluated with the Software Nikon NIS-Elements Basic Research, version 3.0

### RNA isolation and semiquantitative RT-PCR for the normal group

Consecutive sections (slices of 5 μm) of paraffin blocks (n = 6 slices from each block) were scratched from the slides, deparaffinized and treated with Trizol (Invitrogen) to obtain total RNA. Total RNA was used for a DNAase treatment (Invitrogen) followed by a reverse transcription reaction in the presence of MMLV reverse transcriptase (Promega, 200 U/ul) and random hexamer primers (Biodynamics). cDNAs (10 μl) were used to amplify mRNA sequences by a PCR reaction with Go-TaqDNA polymerase (Promega, 5U/ul) and specific primers (1 uM)(Table 1). The first denaturation was performed at 94 °C for 4 min. The successive denaturation steps were performed at 94 °C for 1 min followed by annealing at specific temperature for 1 min and extension at 72 °C for 90 sec. Forty-five cycles were used for each pair of primers. A final elongation of 7 min at 72 °C was used to amplify the transcripts. The amplified cDNA fragments were size-fractioned in 2% agarose gel and visualized in ethidium bromide.The density of bands was quantified with the Scion Image software (NIH).

**Table 1:** Oligonucleotide primers used for PCR amplification of cDNAs obtained after reverse transcription from human testis

Target (Accession number)	Sequence of primer (5'___ 3')	Tm (°C)	Amplified product (bp)
**TGF-β1 (NM_011577)**	F: TCCTGCTTCTCATGGCCA R: CCTCAGCTGCACTTGTAG	55	405
**TGFBRII (NM_009371)**	F: GCGCCAACAACATCAACC R: GGTACTCCTGCAGGTTGC	56	300
**ALK-5 (NM_004612)**	F: GACATCCCTGGTCCATCC R: TCTGGCACAACTCCACTG	55	299
**ALK-1 (NM_000020)**	F: GTCCTGGTTCCGGGAGA R: TTGCTCTTGACCAGCACA	55	294
**Endoglin (NM_000118)**	F: GCATCCTTCGTGGAGCTA R: GACACCTGCATGCCACA	55	300
**β- Actin (AJ312092)**	F: GGATGCAGAAGGAGATCA R: CTAGAAGCATTTGCGGTG	55	193

F: forward; R: reverse

### RNA isolation, semiquantitative and quantitative RT-PCR for infertile patients

Total RNA from human biopsies was extracted with Trizol (Invitrogen) according to the manufacturer's instructions. Total RNA (0.4 ug) was treated with DNAseI (Invitrogen) and used for reverse transcription reaction in a 20 μl reaction using M-MLV reverse transcriptase (Promega, 200 U/ul) and random hexamers primers (Biodynamics).

Reverse-transcribed cDNAs (5 μl) were used to amplify mRNA sequences by a semiquantitative PCR reaction with Go-TaqDNA polymerase (Promega, 5U/ul) and specific primers (1 uM) (Table 1). The first denaturation was performed for 4 min at 94 °C. The successive denaturation steps were performed at 94 °C for 1 min, followed by annealing at specific temperature for 1 min and extension at 72 °C for 90 sec. Thirty cycles were used for each pair of primers. A final elongation of 7 min at 72 °C was used to amplify transcripts. The housekeeping gene used was β-Actin (25 cycles). The amplified cDNA fragments were size-fractionated in 2% agarose gel and visualized by staining with ethidium bromide. The intensity of bands obtained by electrophoresis was evaluated by Scion Image for Windows software. Results are expressed as the ratio between the expression of the specific gene and β-actin.

For quantitative real-time PCR, cDNA was amplified in an ABI PRISM 7700 Sequence Detection System (Applied Biosystems), using SYBR Green Master Mix Reagent (Applied Biosystems). The forward (F) and reverse (R) primers used were: TGF-β1: F: CCCAGCATCTGCAAAGCTC; R: GTCAATGTACAGCTGCCGCA; ALK-1: F: TCTCAGGCCTAGCTCAGATGAT; R: TAGGCTTCTCTGGACTGTTGCT; endoglin: F: CATCCTTGAAGTCCATGTCCTCTT, R: GCCAGGTGCCATTTTGCTT; GADPH: F: TGCACCACCAACTGCTTAGC; R: GGCATGGACTGTGGTCATGAG.

Each primer was used at a concentration of 0.3 μM in each reaction. Cycling conditions were as follows: step 1, 10 min at 95 °C; step 2, 15 sec at 95 °C; step 3, 30 sec at 55 °C; step 4, 30 sec at 60 °C, repeating from step 2 to step 4 40 times. Data from the reaction were collected and analyzed by the complementary computer software (Sequence Detection Software, Applied Biosystems, Version 1.3). Melting curves were run to confirm specificity of the signal. Relative quantification of gene expression was calculated using standard curves and normalized to GADPH in each sample. In order to confirm the best housekeeping gene we evaluated gene expression of *GADPH*, *18 s *and *actin*. *GADPH *showed a lesser CV (3%) than other genes (*18s*: 8.6% and *actin*: 9.3%).

For the assessment of quantitative differences in the cDNA target between samples, the mathematical model of Pfaffl [27] was applied. An expression ratio was determined for each sample by calculating (E_target_)^ΔCt(target)^/(E_GAPDH_)^ΔCt(GAPDH)^, where E is the efficiency of the primer set and ΔCt = Ct _(normalization cDNA) _- Ct _(experimental cDNA)_. The amplification efficiency of each primer set was calculated from the slope of a standard amplification curve of log (ng cDNA) per reaction vs. Ct value (E = 10^-(1/slope)^). Efficiencies of 2 ± 0.1 were considered optimal.

### Statistical analysis

Statistical analysis was performed using Graph Pad Prism (version 3.00 for Windows GraphPad software, San Diego California, USA) for one-way analysis of variance. Data are expressed as mean ± SEM. Tukey's Multiple Comparison or Newman-Keuls tests were used when differences between more than two groups were compared. A p-value of less than 0.05 was considered significant. Pearson's correlation coefficient was also calculated between variants.

## Results

### Hormonal serum levels

Serum levels of testosterone, LH and FSH are shown in Table 2. Testosterone serum levels were within the normal range in the whole population. FSH levels were above the normal range in 12/19. FSH serum concentration in SCO and LCH groups were increased respect to N or H group. LH serum levels were above normal range in 3/19 patients and there were no significant differences between groups.

**Table 2:** Hormonal serum levels

Pathology	FSH (mIU/ml)	LH (mIU/ml)	T (pmol/ml)
SCO (n = 6)	16.1 ± 1.8 **^a,b^**	7.8 ± 1.5	14.5 ± 2.1
H (n = 8)	5.3 ± 0.6 **^a^**	4.3 ± 0.3	18.4 ± 1.7
LCH (n = 5)	26.8 ± 6.7 **^b^**	7.1 ± 1.7	17.0 ± 2.4
N (n = 4)	3.8 ± 1.1 **^a^**	5.2 ± 0.3	21.2 ± 1.7

SCO: Sertoli cell-only Syndrome; H: Hypospermatogenesis; LCH: Leydig Cell Hyperplasia. N: normal. Values represent the mean ± SEM. Tukey's Multiple Comparison was done between groups. Different letters indicate significant differences between groups (p < 0.005).

### Immunohistochemistry of TGF-β1, ALK 1 and 5 and Smad proteins in normal testicular tissue

**Figure 1:**
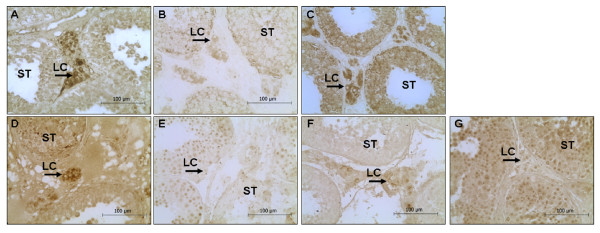
**TGF-β1 system in normal testes**. Sections of normal testes showing immunohistochemistry of TGF-β 1 (Panel A); ALK-5 (Panel B); ALK-1 (Panel C), Smad 2/3 (Panel D), p-Smad 2/3 (Panel E); Smad 1/5 (Panel F); p-Smad 1/5 (Panel G). Magnification 400×.

The immunostaining of TGF-β1 was clearly detected in Leydig cells of normal tissue (Figure 1 Panel A). A weak staining was observed for ALK-5, while a strong staining was observed for ALK-1 in Leydig cells (Figure 1 Panels B and C, respectively).

Smads 2/3 (Figure 1 Panel D) were detected in the cytoplasm of Leydig cells but their phosphorylated forms (Figure 1 Panel E) were located in the nucleus of Leydig cells. In the seminiferous tubules (ST), total Smad 2/3 (Figure 1 Panel D) was observed in the nucleus of germinal line, whereas p-Smad 2/3 was not detected (Figure 1 Panel E).

Leydig cells showed a significant staining for Smad 1/5 in the cytoplasm but a weak nuclear staining for p-Smad 1/5 (Figure 1 Panels F and G, respectively). In the ST, Smads 1/5 (Figure 1 Panel F) were observed in the cytoplasm and nucleus of spermatids, spermatogonia and spermatocytes, whereas their phosphorylated form (Figure 1 Panel G) was clearly detected in the nucleus of those cells (Figure 1 Panel F).

### Immunohistochemistry of TGF-β1, ALK-1 and 5 and endoglin in testicular pathologies

**Figure 2:**
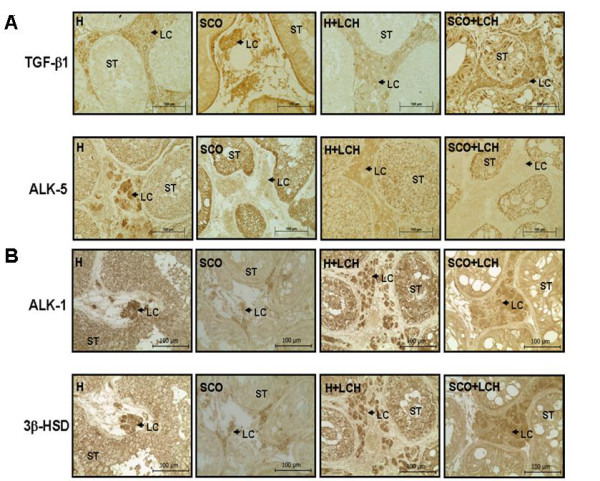
**Localization of TGF-β1, ALK-5 and ALK-1 in testicular pathologies**. Panel A: Immunostaining of TGF-β1 and ALK-5 in testicular pathologies. Panel B: Immunostaining in serial sections of ALK-1 and 3β-hydroxysteroid dehydrogenase (3β-HSD) in testicular pathologies. H: Hypospermatogenesis, SCO: Sertoli cell-only Syndrome, LCH: Leydig Cell Hyperplasia, ST: Seminiferous Tubules, LC: Leydig cell. Magnification 400 x.

Specific immunohistochemical analysis revealed the presence of TGF-β1 in the cytoplasm of testicular Leydig cells of all patients (Figure 2). The intensity of TGF-β1 immunostaining in patients with LCH was higher than that in patients without LCH (SCO vs SCO+LCH: 2.7 fold, p < 0.01; H vs H+LCH: 3.8 fold, p < 0.05).

ALK-5 immunostaining (Figure 2) was weak in Leydig cells in pathological biopsies, but strong in ST of SCO patients. Co-localization of ALK-1 and 3β-HSD, a marker of Leydig cells, was evaluated in consecutive sections by immunohistochemistry. ALK-1 immunostaining (Figure 2) was stronger in Leydig cells of LCH patients than in patients without LCH (SCO vs SCO+LCH: 2.1 fold, p < 0.01; H vs H+LCH: 1.7 fold, p < 0.05).

**Figure 3:**
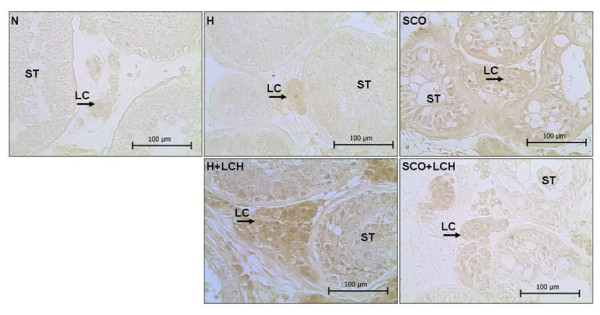
**Immunolocalization of endoglin**. N: normal, H: Hypospermatogenesis, SCO: Sertoli cell-only Syndrome, LCH: Leydig Cell Hyperplasia, ST: Seminiferous Tubules, LC: Leydig cell. Magnification 400 x.

Endoglin was present in SCO or H with LCH group but not detected in normal tissue (Figure 3).

### Immunohistochemistry of Smad proteins in human pathologies

**Figure 4:**
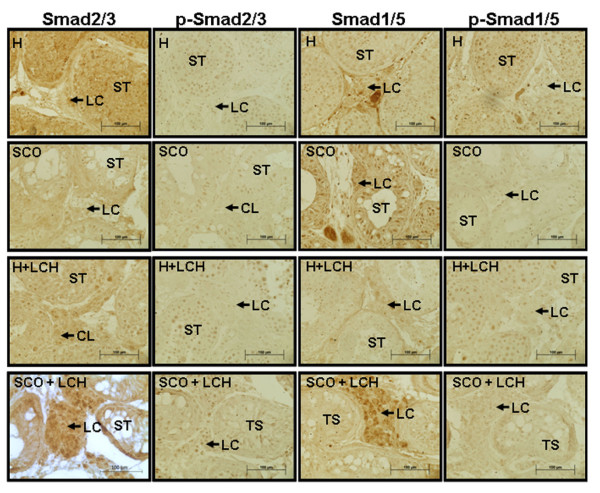
**Immunolocalization of Smad 2/3, p-Smad 2/3, Smad 1/5 and p-Smad 1/5 in human pathologies**. ST: Seminiferous Tubules, LC: Leydig cell. Magnification 400 x

Total Smads 2/3 and their activated phosphorylated forms were detected in SCO, H and LCH patients. These proteins were observed mainly in SCO patients that presented LCH (Figure 4).

Patients of the H group presented Smad 1/5 staining in the cytoplasm of Leydig cells and in the nucleus of spermatids and spermatogonias. However, p-Smad 1/5 signaling was detected in the cytoplasm and nucleus of Leydig cells and in the nucleus of all cells of the germ line.

Patients of the SCO group showed a clear staining of Smad 1/5 in the cytoplasm of Leydig cells that was more evident in SCO patients with LCH. The activated form, p-Smad 1/5, was observed in the nucleus of Leydig cells as well as in some Sertoli cells.

### *TGF-β1, TGFBRII, ALK-1, ALK-5*, and *endoglin *expression in normal tissue

**Figure 5:**
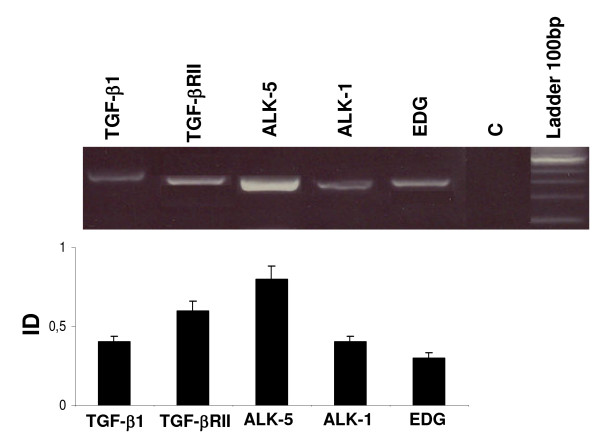
**Gene expression of *TGF-β1, TGFBRII, ALK-5, ALK-1 *and e*ndoglin *(EDG) in normal testicular tissue**. Results are represented with an arbitrary scale and expressed as the ratios between the expressions of the specific gene and *β-actin *(integrated density, ID). Data are plotted as the mean ID ± SEM. Tukey's Multiple Comparisons were done between groups. Different letters indicate significant differences (p< 0.05)

The expression of *TGF-β1, TGFBRII, ALK-1, ALK-5 *and *endoglin *in normal testicular tissue is shown in Figure 5. A clear band for the expected molecular weight was observed for each cDNA analyzed, revealing the presence of these components of the TGF-β1 system in normal tissue.

### *TGF-β1, TGFBRII, ALK-1 ALK-5 *and *endoglin *expression in pathological tissue

**Figure 6:**
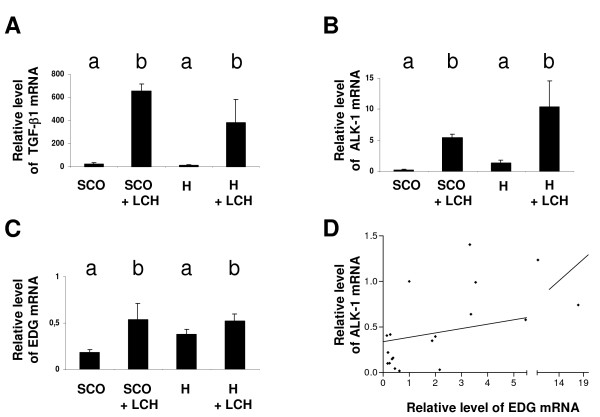
**Gene expression of *TGF-β1*, *ALK-1 *and e*ndoglin *in pathological tissue**. Quantitative gene expression of *TGF-β1 *(Panel A), *ALK 1 *(Panel B) and *Endoglin *(EDG) (Panel C) in pathological tissue. Different letters indicate significant differences (p < 0.05). Results are represented with an arbitrary scale and expressed as the ratios between the expressions of the specific gene and GADPH (integrated density, ID). Data are plotted as the mean ID ± SEM. Newman-Keuls Multiple Comparison was done between groups. Different letters indicate significant differences (p< 0.05). Panel D: Correlation between ALK-1 and Endoglin gen expression mRNA. (Pearson coefficient, r = 0. 4915; p < 0.05)

Semiquantitative PCR of the TGF-β1 system showed that neither *TGFBRII *nor *ALK-5 *exhibited significant differences between the experimental groups, but the mRNA expression of *TGF-β1, ALK-1 *and *endoglin *were significantly increased in patients with LCH respected to the other groups (data not shown). Since the proliferative action of *TGF-β1 *depends only of *ALK-1 *and *endoglin*, and not of *TGFBRII *and *ALK-5*, we further performed a quantitative PCR to confirm these results. *TGF-β1, ALK-1 *and *endoglin *mRNA clearly showed a significant increase in testicular biopsies from LCH group (independently of whether LCH derived from SCO or H) than in SCO or H (Figure 6 Panels A, B and C) (p< 0. 05).

Statistical analysis showed a positive correlation between *ALK-1 *and *endoglin *expression (r = 0.4915 Pearson; p < 0.05) (Figure 6 Panel D) independently of the pathology.

## Discussion

Testicular LCH is a benign condition in which the hyperplasic cells infiltrate between ST [28]. Adult patients with LCH are usually infertile. No cause can account for all cases of LCH. In this context, the study of some factors involved in the proliferation of Leydig cells might contribute to the understanding of this pathology. Some reports [28] have suggested that an increase in serum LH can result in LCH. However, in the present study, the serum LH levels of patients were within the normal range. Clegg et al. [3] proposed that an alteration of the production of paracrine growth factors could result in growth stimulation without major effects on the circulating LH levels. FSH levels are increased in SCO and LCH patients. These results are in agreement with those of other authors [1,29], who have described that the increased FSH levels in SCO patients are due to the lack of inhibin, a main regulator of FSH secretion by a negative feedback mechanism.

The presence of TGF-β1, TGFBRII and TGFBRI has long been described in human testis [21,22]. In the present work, we analyzed the TGF-β1 system in SCO and H with or without LCH. The histochemistry of TGF-β1 showed the presence of this peptide in the cytoplasm of Leydig cells from patients with SCO, H and LCH. The testicular staining of TGF-β1 was stronger in patients that present LCH than in patients with normal Leydig cell morphology.

Dobashi et al.[21] demonstrated by immunohistochemistry that the presence of extracellular and intracellular TGF-β1 is higher in SCO patients than in normal controls and determined that the serum level of this cytokine is higher in this group of patients. In the present study, we also detected an increase in TGF-β1 immunostaining in H patients with LCH. In addition, mRNA molecular analyses showed that gene expression of TGF-β1 resembles that observed by immunohistochemistry studies; i.e., the mRNA of this cytokine is higher in LCH than in SCO or H patients.

Several authors have detected TGBRI, however, no information about differential expression of ALK-1 and 5 in human testicular tissues are published. Zhang et al. [22] have demonstrated, in normal men, that the staining for TGFBRI protein is stronger in the testicular interstitium, i.e. Leydig cells, weaker in the Sertoli cells, and not detectable in spermatogonia and primary spermatocytes. According to the results obtained with antibody used by these authors, the ALK-5 receptor was the subtype detected. In our study, ALK-5 gene expression did not show significant differences between groups. Konrad et al. [30] have described that TGF-β1 exerts an apoptotic effect. Thus, we could speculate that the absence of germinal cells is a consequence of the action of TGF-β1, via ALK-5 receptor, on seminiferous tubules.

In the present study, we observed that ALK-5 is immunohistochemically localized in Leydig cells and show a weak staining in ST of normal men. However, SCO patients showed a strong staining in this testicular compartment. On the other hand, ALK-1 staining is sparsely present in normal Leydig cells but stronger in LCH. ALK-1 gene expression is also higher in testes from LCH than in those from SCO or H. To our knowledge, this is the first report showing the cellular localization of ALK-1 in normal and pathological testes.

Suszko and Woodruff [31] have pointed out that the availability and activity of the receptor can influence a particular signaling pathway. These authors considered that the receptor activity and the length of their active state are also crucial to facilitate a particular cellular response. In this context, it is important to remark that the TGFBRI receptor has been associated with cholesterol-rich membrane microdomains, known as caveolae, and consequently, with the suppression of TGF-β1-mediated phosphorylation of Smad2 [32,33].

Immunohistochemistry showed a clear staining of endoglin in Leydig cells of the LCH group but not in those of normal tissue. On the other hand, we detected the expression of *endoglin *mRNA in normal and pathological tissues, and found that it was higher in LCH patients than in other groups.

The lack of stain in normal tissue might be due to the different sensitivities of the two methods (PCR and histochemistry). Besides, it is possible that the mechanism of endoglin mRNA transduction into protein in normal tissue was diminished. Different mechanisms of post-transcriptional control have been described: elongation and termination, alternative RNA splicing, 3'cleavage and polyadenylation, nuclear export, regulation of stability and translation of mRNAs and regulation of the subcellular location of specific mRNAs.

Statistical studies revealed a correlation between *ALK-1 *and *endoglin *gene expression. Since in endothelial cells ALK-1 and endoglin have been involved in TGF-β1 signaling leading to proliferation [20], we could speculate that these components of the TGF-β1 system might participate in the proliferation of Leydig cells observed in LCH patients.

Several studies have suggested that Smad proteins are the intracellular mediators of TGF-β signaling [34-36]. These transcription factors translocate to the nucleus upon ligand stimulation. There is scarce information about Smad proteins in human testes since the limitation of tissue access precludes knowledge of the characterization of TGF-β1 signaling components. Thus, we aimed to analyze the presence of these proteins in testicular biopsies from our patients. We observed that total Smad 2/3 and 1/5 are present in Leydig cells, mainly in the LCH group. Moreover, both phosphorylated isoforms were detected.

Sun et al. [37] observed an increase in Smad2 expression in SCO syndrome compared to normal men, but did not report the presence or absence of LCH. Narula et al. [16] have described LCH, apoptosis of germ cells and spermatogenic arrest in transgenic mice overexpressing the common factor Smad4.

In the mouse postnatal testis, mRNA and protein Smad1 have been detected in spermatocytes and round spermatids [38]. Smad 5 protein has been reported only in prepubertal mouse testes, being restricted to spermatogonia [39]. Reports of Smad 4 show a ubiquitous localization in mouse testes [39,40], whereas it seems to have a specific Leydig cell localization in the rat [40].

Recently, we have described the hormonal regulation of TGF-β1 system in an experimental model [41] and that the *in vitro *effect of TGF-β1 on mouse Leydig cell induces Smad 1/5 phosphorylation and proliferation markers (Bax/Bcl-2 ratio, PCNA) [41].

Expression of different receptor subtypes would allow to Leydig cells to mount an appropriate response to extracellular stimulation by the differential presence and phosphorylation of Smad proteins, leading to activation of distinct target genes.

Whether the high expression of the TGF-β1 system observed in patients with LCH is a cause or a consequence of this pathology should be analyzed. Unfortunately, physiological studies can not be developed in testicular biopsies. Thus, further studies using suitable animal experimental models should be performed in order to investigate the role of the TGF-β1 system in Leydig cells. In this context, we analyzed that the *in vivo *intratesticular administration of this cytokine stimulates Leydig cell hyperplasia/hypertrophy [42].

## Conclusions

Together, the present study and our previous ones [41,42] seem to indicate that the LCH observed in human biopsies are likely to be, at least in part, due to the increase in the TGF-β1 system. These studies might be of great importance to apply this knowledge to key outcomes in male reproductive health.

## Competing interests

The authors declare that they have no competing interests.

## Authors' contributions

SIG-C and RSC were responsible for designing and coordinating the study as well as for data interpretation and writing of the manuscript. CG performed the experiments.

MEM and MBF were involved in data collection and data analysis of the study.

CT, EP and OL participated in the clinical part of the study. RP performed the morphological characterization of the biopsies All authors read and approved the final manuscript.
